# Supplementary material for: Digital Form for Assessing Dentists’ Knowledge about Oral Care of People Living with HIV
Source: Int J Environ Res Public Health. 2022 Apr 21;19(9):5055. doi: 10.3390/ijerph19095055 (PMC9103845; doi:10.3390/ijerph19095055)
Supplement: Supplementary file 1 [file ijerph-19-05055-s001.zip › ijerph-1616826-supplementary.pdf]

Supplementary Material: All queries from block 4.

1) No seu conhecimento técnico AIDS (Acquired Immunodeficiency Syndrome) ou SIDA (Síndrome da Imunodeficiência Adquirida) são as mesmas coisas que HIV (Vírus da Imunodeficiência Humana)? *

- ◯ Sim
- ◯ Não
- ◯ Talvez

2) No seu conhecimento técnico vossa senhoria saberia como o HIV age no organismo? *

- ◯ Sim
- ◯ Não
- ◯ Talvez

Encarecidamente solicitamos que justifique sua respostas com suas próprias palavras.

Sua resposta

3) No seu conhecimento técnico quais são os possíveis meios de transmissão do HIV? Assinale todas as opções de meios de transmissão? *

- [ ] Relações sexuais desprotegidas
- [ ] Compartilhamento de produtos de higiene pessoal (escovas de dente e sabonete)
- [ ] Transfusão sanguínea com sangue contaminado
- [ ] Compartilhamento de pratos, talheres e copos
- [ ] Compartilhamento de agulhas contaminadas
- [ ] Acidentes laborais com materiais perfuro-cortantes
- [ ] Contato em mucosa ou feridas com fluidos corpóreos como sangue, suor ou lágrimas
- [ ] Contato por aperto de mão ou abraço
- [ ] Via aerossol no consultório odontológico
- [ ] Transmissão vertical (Mãe-feto)

4) No seu conhecimento técnico quais destas lesões orais podem ser associadas a pacientes vivendo com HIV/AIDS e servir para o diagnóstico sugestivo no consultório odontológico? *

- [ ] Candidíase oral
- [ ] Condiloma acuminado
- [ ] Gengivite necrosante
- [ ] Gengivo-estomatite herpética
- [ ] Eritema Gengival Linear
- [ ] Sarcoma de Kaposi
- [ ] Xerostomia (boca seca)
- [ ] Todas as alternativas acima
- [ ] Hiperplasia epitelial focal
- [ ] Queilite angular
- [ ] Estomatite necrosante
- [ ] Leucoplasia pilosa
- [ ] Periodontite necrosante
- [ ] Ulcerações aftosas

5) No seu conhecimento técnico quais destes sinais e sintomas podem ser observados, durante a anamnese odontológica, em pacientes com AIDS (Síndrome da Imunodeficiência Adquirida)?
*

- [ ] Gripe prolongada
- [ ] Febre
- [ ] Tosse seca
- [ ] Mal-estar
- [ ] Dor de garganta
- [ ] Náusea
- [ ] Vômito
- [ ] Linfonodomegalia (inchaço dos gânglios linfáticos)
- [ ] Fadiga
- [ ] Perda de peso sem motivos
- [ ] Sudorese noturna (suor noturno)
- [ ] Diarreia persistente
- [ ] Perda de apetite
- [ ] Todas as alternativas acima

6) No caso de um paciente apresentar sinais e sintomas médicos e odontológicos condizentes com infecção pelo HIV (Vírus da Imunodeficiência Humana), vossa senhoria saberia quais exames laboratoriais solicitar para o diagnóstico conclusivo e como interpretar estes exames laboratoriais? *

- Sim
- Não
- Talvez

Se respondeu sim na pergunta acima, por favor, exemplifique um local a qual vossa senhoria encaminharia o paciente para fazer os exames.

Sua resposta

7) Ainda tomando como exemplo a questão 6, no caso do exame ter resultado positivo para o HIV (Vírus da Imunodeficiência Humana), vossa senhoria saberia para qual(is) serviço(s) deve-se encaminhar o(a) referido(a) paciente? *

- Sim
- Não
- Talvez

Se respondeu sim na pergunta acima, por favor, exemplifique um local a qual vossa senhoria encaminharia o paciente para tratamento no local adequado.

Sua resposta

8) Ainda tomando como exemplo a questão 6, no caso do exame ter resultado negativo para o HIV (Vírus da Imunodeficiência Humana), vossa senhoria solicitaria um segundo exame anti-HIV após 30 dias da exposição ao vírus? *

- Sim
- Não
- Talvez

Encarecidamente solicitamos que justifique sua respostas com suas próprias palavras.

Sua resposta

9) Ao atender pacientes vivendo com HIV/AIDS, vossa senhoria faz alguma mudança ou alteração no uso dos vossos EPI's (Equipamento de Proteção Individual) ou na esterilização dos instrumentais após atender esses paciente? *

- Sim
- Não
- Talvez

Se respondeu sim na pergunta acima, por favor, exemplifique essas mudanças.

Sua resposta

10) A Terapia Antirretroviral (TARV) vem sendo o tratamento de escolha para pacientes vivendo com HIV/AIDS o que vem permitindo aos mesmos(as) a conviverem com o HIV, geralmente esses pacientes apresentam uma polifarmácia com os seus conhecimentos técnicos, vossa senhoria teria dúvidas ou receios de realizar prescrição medicamentosa devido a esta polifarmácia? *

- Sim
- Não
- Talvez

11) Em caso de atendimento não invasivo ou controle de higiene em pacientes vivendo com HIV/AIDS, vossa senhoria faria alguma alteração na sua conduta clínica, ou uso de EPI's ou limpeza do consultório devido ao possível contato com saliva e/ou sangue, além do aerosol produzido durante o procedimento? *

- Sim
- Não
- Talvez

12) Em casos com indicação para procedimentos invasivos em pacientes vivendo com HIV/AIDS, vossa senhoria mudaria seu protocolo clínico ou até mesmo evitaria realizar procedimentos invasivos cirurgias devido ao risco de contato com fluidos do paciente ou acidentes com instrumentos perfurocortantes? *

- Sim
- Não
- Talvez

13) Vossa senhoria teria alguma dúvida, incerteza ou insegurança frente o atendimento e tratamento de pacientes vivendo com HIV/AIDS que queira compartilhar com os pesquisadores?

Sua resposta
